# Aging Equally? Employing Active Aging Policy in the United Kingdom

**DOI:** 10.1093/geroni/igab046.1662

**Published:** 2021-12-17

**Authors:** Liam Foster

**Affiliations:** University of Sheffield, Sheffield, England, United Kingdom

## Abstract

The UK’s responses to the challenges of ageing have largely focused on productivist notions of active ageing, with more comprehensive responses tending to be reactive and largely remedial. This presentation will show that productivist policies, often characterised by individual responsibility, including raising the retirement age, restricting access to early retirement, and providing a stronger link between pension benefits and contributions, have incentivised remaining in the labour market. These strategies have been justified in the context of ageing populations and increasing pension costs. However, opportunities to extend working lives have not been experienced equally. In practice most policies are gender blind. Furthermore, a more comprehensive approach to active ageing in the UK needs a collective emphasis to mobilise a wide range of societal resources, underpinned by a commitment to public welfare, which is highly problematic under neo-liberalism. Therefore, a comprehensive approach to ageing in the UK requires a substantial ideological shift.

